# Targeted sequencing reveals *TP53* as a potential diagnostic biomarker in the post-treatment surveillance of head and neck cancer

**DOI:** 10.18632/oncotarget.11196

**Published:** 2016-08-11

**Authors:** Joost H. van Ginkel, Wendy W.J. de Leng, Remco de Bree, Robert J.J. van Es, Stefan M. Willems

**Affiliations:** ^1^ Department of Oral and Maxillofacial Surgery, University Medical Center Utrecht, Utrecht, The Netherlands; ^2^ Department of Pathology, University Medical Center Utrecht, Utrecht, The Netherlands; ^3^ Department of Head and Neck Surgical Oncology, University Medical Center Utrecht, Utrecht, The Netherlands

**Keywords:** head and neck cancer, mutations, next-generation sequencing, *TP53*, diagnostic biomarkers

## Abstract

Head and neck squamous cell carcinomas (HNSCC) form a large heterogeneous group of tumors and have a relatively poor outcome in advanced cases. Revealing the underlying genetic mutations in HNSCC facilitates the development of diagnostic biomarkers, which might lead to improved diagnosis and post treatment surveillance. We retrospectively analyzed mutational hotspots using targeted next-generation sequencing (NGS) of 239 HNSCC tumor samples in order to examine the mutational profile of HNSCC. Furthermore, we assessed prevalence, co-occurrence, and synonymy of gene mutations in (matched) tumor samples. *TP53* was found mutated the most frequent with mutation rates of up to 83% in all tumors, compared to mutation rates of between 0 and 21% of *CDKN2A*, *PIK3CA*, *HRAS*, *CDK4*, *FBXW7* and *RB1*. Mutational co-occurrence predominantly existed between *TP53* and *PIK3CA*, *TP53* and *CDKN2A*, and *HRAS* and *PIK3CA*. Mutational synonymy between primary tumor and associated metastasis and recurrence was present in respectively 88% and 89%. *TP53* mutations were concordantly mutated in 95% of metastases and in 91% of recurrences. This indicates *TP53* mutations to be highly prevalent and concordant in primary tumors and associated locoregional metastases and recurrences. In turn, this provides ground for further investigating the use of *TP53* mutations as diagnostic biomarkers in HNSCC patients.

## INTRODUCTION

Head and neck squamous cell carcinomas (HNSCC) originate from various anatomic sites in the upper aerodigestive epithelium, i.e. the oral and (para) nasal cavities, pharynx and larynx. HNSCC is the sixth most common cancer worldwide with an estimated incidence of 4.8% of all malignancies in the entire body [1]. Etiologically, these tumors roughly fall into two main distinct groups: tumors induced either by tobacco smoking or chewing (e.g. betelnut) and alcohol abuse, or by viral infection with Human Papilloma Virus (HPV) or Epstein Barr Virus (EBV). Alcohol and/or tobacco induced HNSCCs are strongly associated with somatic mutations in tumor suppressor genes (TSG) such as *TP53, CDNK2A, PTEN*, and oncogenes (OG) such as *HRAS* and *PIK3CA* [2–5]. *TP53* inactivating HPV oncoproteins E6 and E7 are the main cancer initiators in an increasing number of oropharyngeal squamous cell carcinoma (OPSCC) cases [6]. The overall survival of advanced cases still remains poor. This is especially true for HPV-negative tumors as compared to HPV-positive tumors [7, 8]. Furthermore, the mutational profile of HNSCC appears to significantly affect its disease course and prognosis [9–12]. Although disease outcome of HNSCC depends on multiple levels of disease processes (e.g. pathogenesis, molecular characteristics, and TNM-stage), estimation of its prognosis is still largely based on the tumor stage at clinical presentation and relapse after initial treatment. Furthermore, possibility of successful salvage treatment is largely dependent on early detection and the extent of the locoregional disease [13–16]. This underlines the need to explore new possibilities for improving diagnostics on a molecular level. The use of diagnostic biomarkers could enable detection of tumor specific mutations in order to monitor tumor response after treatment with curative intent. Ultimately, this might improve treatment outcome of HNSCC patients, while avoiding unnecessary (over) treatment and its associated morbidity and accompanying hindrance to the patient.

Technological advances over recent decades have improved the understanding of tumor genetics. Consequently, targeted profiling of tumor genetics is gradually shifting from an experimental setting towards its use in routine clinical practice in fields such as breast and lung oncology [17]. Although no common ground exists yet for the use of biomarkers in clinical decision making for HNSCC patients, evidence for future use is arising [18–20]. As previous studies have shown, *TP53* is highly prone to loss of heterozygosity. This leads to the presence of inactivating non-hotspot mutations of *TP53* that occur early in HNSCC carcinogenesis [21–24]. Subsequently, subclonal cells from the primary tumor either proliferate towards metastases or locally reside after treatment and develop into recurrences. These clonal expansions are likely to contain the early onset mutations found initially in the primary tumor [25–27]. By using dedicated and clinically accessible gene panels based on NGS, these mutations can reliably be detected and selected as targets. Circulating tumor DNA (ctDNA) released by clonal expansion cells contain these targets and could be quantified using minimally invasive blood samples, as there appears to be a relation between ctDNA plasma concentrations and tumor burden [28–31].

However, the significance of ctDNA in correlation with actual tumor burden and/or tumor growth still needs to be proven for HNSCC patients. This requires research on the identification of early driver gene mutations of HNSCC tumors. Therefore, we retrospectively analyzed a large dataset of sequenced HNSCCs, to map their mutational profile and to explore TP53 and possible other genes as potential diagnostic biomarkers in HNSCC.

## RESULTS

### Patient and tumor characteristics

A total of 110 patients accounted for 239 tumor samples that remained for analysis. Eighty (73%) patients were male. Of all patients, 76 (69%) had a history of smoking tobacco and 67 (61%) had a history of alcohol consumption. Eleven (10%) patients never used tobacco or alcohol. For 18 patients, either or both tobacco smoking and alcohol use was unknown. Table 1 summarizes patient and tumor characteristics of our study group.

**Table 1:** Patient and tumor characteristics

Patients	110
Tumor samples	239
Mean age, years (range)	66 (45-90)
Sex	n (%)
Male Female	80 (73) 30 (27)
Smoking history	n (%)
Yes No Unknown	76 (69) 20 (18) 14 (13)
Alcohol use	n (%)
Former/active Never Unknown	67 (61) 32 (29) 11 (10)
Clinical stage*	n (%)
T1-2 T3-4 N0 N1-2 Unknown	100 (70) 44 94 (67) 50 5
Primary tumor sites	n (%)
Oral cavity Oropharynx Hypopharynx Larynx Miscellaneous	53 (36) 37 (25) 16 (11) 28 (19) 14 (9)
Tumor subtype	n (%)
Primary Recurrence Metastasis	148 (62) 29 (12) 62 (26)
HPV status**	n (%)
Positive Negative	4 (12) 29 (88)

* Included all primary and secondary primary tumors

** All positive tumors were OPSCCs

Of all 239 tumor samples, 148 (62%) were primary site squamous cell carcinomas. Of the primary tumor samples, 53 (36%) originated from the oral cavity, 37 (25%) from the oropharynx, 16 (11%) the hypopharynx, and 28 (19%) from the larynx. Fourteen (9%) primary tumors originated from miscellaneous sites (i.e. nasopharynx and upper esophagus and trachea). Of the 37 OPSCCs, 33 were tested for HPV-status. Only four (12%) samples proved HPV-positive. The remaining 91 out of 239 tumor samples comprised of 29 (12%) recurrences and 62 (26%) metastases. The latter could be subdivided into 38 (62%) nodal metastases and 23 (38%) distant metastases in the lung, liver, bones, or skin.

### Mutational analysis

**Figure 1:**
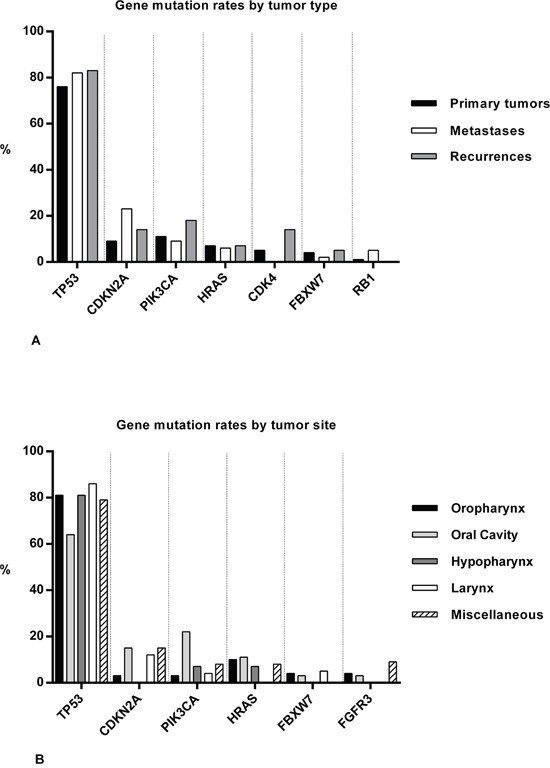
Summary of gene mutations in HNSCC samples Bar charts showing mutation rates (%) of genes in all tumor samples ordered by tumor type **A.**, and in 148 primary tumor samples ordered by HNSCC sites **B.**

Sequencing was based on Cancer Hotspot Panel v2 (CHPv2) for 160 tumor samples, OncoAmp Panel v2 (OAPv2) was used for 40 samples, and Cancer Hotspot Panel v2+ (CHPv2+) was used for sequencing of 11 samples (Table 2). Additional Sanger sequencing was performed in 28 cases, in which NGS failed due to insufficient DNA quantity. NGS of the exons that are included in the three different gene panels (as described in our method section) yielded mutations in 26 different genes. No mutations were detected in *ABL1, MYD88, NOTCH1, AKT1, ARAF, GNAS, GNA11, NRAS, PDGFRA, CALR, CDH1, IDH1, PTPN11, RET, SMO, SRC, STK11, VHL, MLH1, MPL, JAK3, JAK2, IDH2, CRAF, CSF1R, CTNNB1*, and *EZH2*. *TP53* had the highest mutation rates in recurrences (83%), metastases (82%) and primary tumor samples (76%). These rates compared to mutation rates of *CDKN2A*, *PIK3CA*, *HRAS*, *CDK4*, *FBXW7* and *RB1* of between 0 and 21% (Figure 1A). Furthermore, *TP53* was found mutated most frequently in OPSCC (81%), OSCCs (64%), HPSCCs (81%), LSCC (86%), and in miscellaneous tumors (79%). On average, other frequently mutated genes in HNSCC sites were *PIK3CA* (11%)*, CDKN2A* (10%), *HRAS* (8%)*, FGFR3* (3%) and *FBXW7* (3%) (Figure 1B). In the 130 successfully sequenced primary tumor samples, no mutations were detected in 21 (16%) samples. In one of the HPV-positive tumor samples, a single *TP53* mutation (c.225-35G>C) was found. In the other HPV-positive samples, no mutations were detected. Full range of mutated genes with prevalence rates for all subgroups is provided in Supplementary Tables S1 and S2.

**Table 2:** Used gene panels for sequencing of tumor samples

	CHPv2	CHPv2+	OAv2	Sanger	Total
Primary	99	9	22	18	148
*OPSCC* *OSCC* *HSCC* *LSCC* *Misc*	*24* *32* *11* *21* *11*	*3* *6* *-* *-* *-*	*4* *8* *4* *4* *2*	*6* *7* *1* *3* *1*	*37* *53* *16* *28* *14*
Recurrence	19	2	7	1	29
Metastasis	42	-	11	9	62
Total	160	11	40	28	239

**Figure 2:**
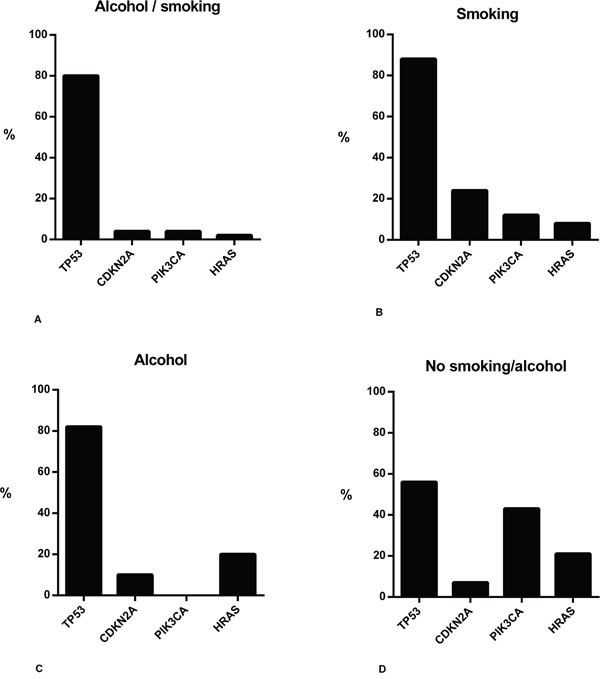
Summary of gene mutations in HNSCC samples of patients with or without a history of smoking and/or alcohol use Bar charts showing mutation rates of primary tumor samples related to a history of smoking and alcohol use **A.**, exclusively smoking **B.**, exclusively alcohol use **C.**, and samples not related to a history of smoking and alcohol use **D.**

The 92 patients with a history of alcohol use and/or smoking accounted for 121 primary tumor samples in total. *TP53* was sequenced in all 121 samples. *CDKN2A*, *PIK3CA*, and *HRAS* were sequenced in 110 of 121 samples (Table 3). In all tumor samples of patients with or without a history of smoking and alcohol use, highest mutation rates were found in *TP53*, *CDKN2A*, *PIK3CA*, and *HRAS*. Mutation rates for *TP53* were between 56 and 88%, for *CDKN2A* between 4 and 24%, for *PIK3CA* between 0 and 43%, and for *HRAS* between 2 and 21%. Overall, tumors exclusively related to a history of smoking had the highest mutation rates compared to the other subgroups (Figure 2A-2D).

**Table 3:** Prevalence of gene mutations in alcohol and/or smoking related tumor samples

	Smoking/alcohol		Non- smoking/alcohol		Smoking		Alcohol		Total	
	No.	(%)	No.	(%)	No.	(%)	No.	(%)	No.	(%)
*TP53*	55/69	(80)	9/16	(56)	22/25	(88)	9/11	(82)	96/121	(79)
*CDKN2A*	2/60	(4)	1/14	(7)	6/25	(24)	1/10	(10)	11/110	(10)
*PIK3CA*	2/60	(4)	6/14	(43)	3/25	(12)	0/10	-	11/110	(10)
*HRAS*	1/60	(2)	3/14	(21)	2/25	(8)	2/10	(20)	8/110	(7)

### Mutational co-occurrence

**Figure 3:**
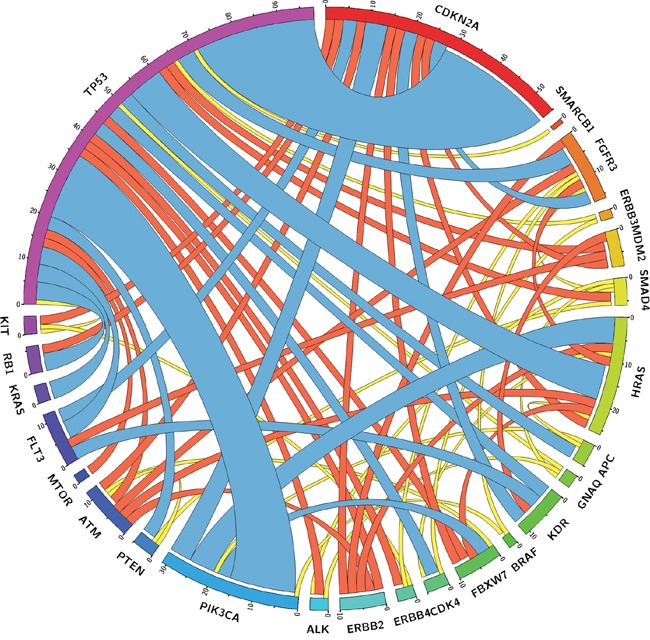
Circle plot illustrating co-mutations between genes found in HNSCC samples Outer bars showing the absolute total amount of co-mutations found for genes entitling the bars. The curved ribbons inside the circle depict absolute number of co-mutations with the genes they are connected with (ribbon thickness corresponds with number of co-mutations). Ribbons are color coded by quartiles Q1 (yellow), Q2 (red), and Q3 (blue).

All tumor samples accounted for 171 mutational co-occurrences of two genes within one tumor sample. Co-occurrences were mostly found between *TP53* and *CDKN2A* (16%), *TP53* and *PIK3CA* (9%), *TP53* and *HRAS* (5%), *PIK3CA* and *HRAS* (4%), and *PIK3CA* and *CDKN2A* (3%), as shown in Figure 3 and Supplementary Table S3.

### Mutational synonymy

**Figure 4:**
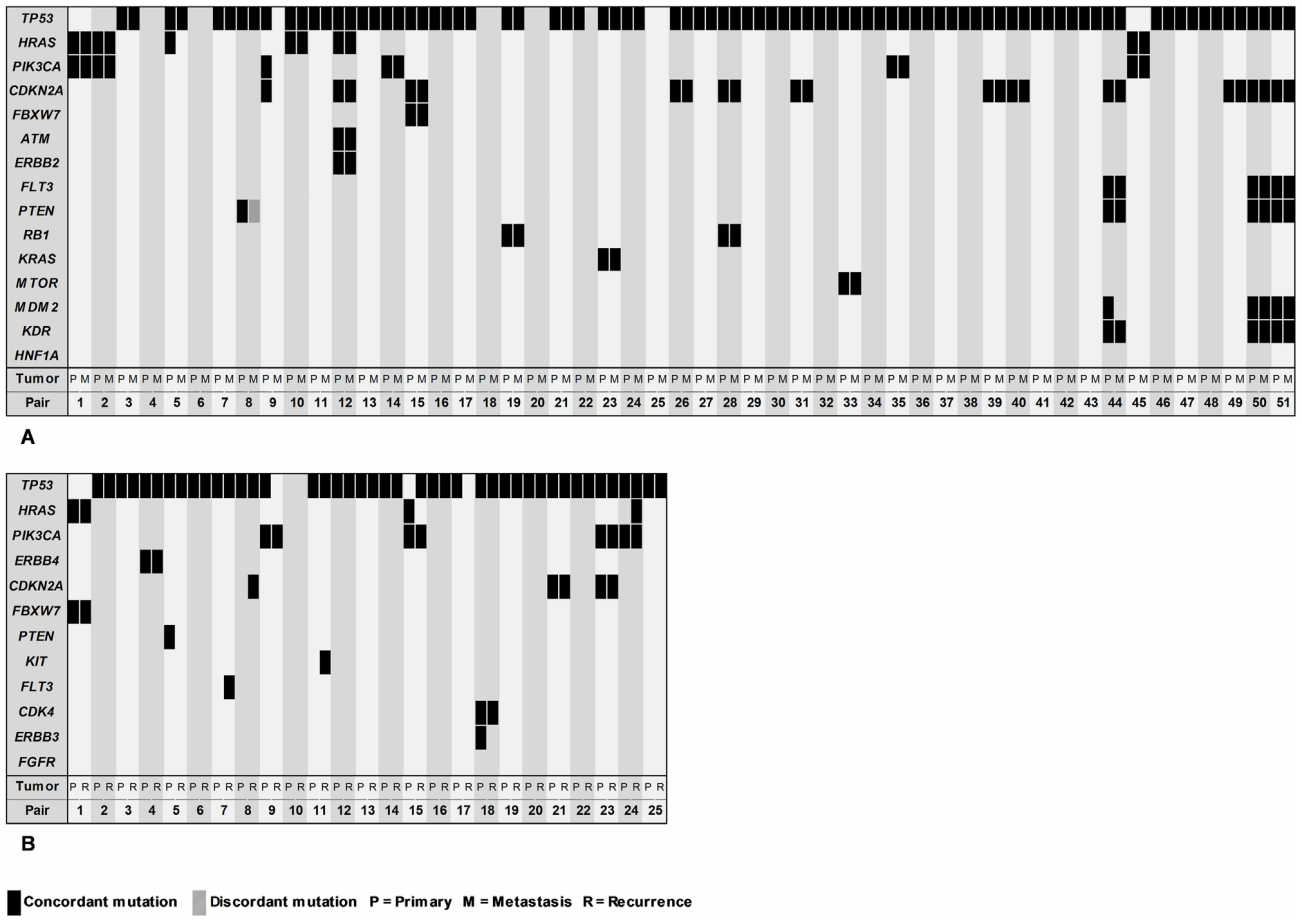
Block chart showing the mutational synonymy within tumor pairs Mutational profiles of primary tumors (P) on the left side of vertical bars, compared to metastases (M) in **A.**, and recurrences (R) in **B.** on the right side of the bars. Metastases and recurrences were either matched to a primary tumor or a second primary tumor.

Sequencing data of the associated (second) primary tumor were available for 51 of the 62 regional and distant metastases (the remaining 11 metastases were either associated to unsequenced primary tumors or were recurrent tumors only). By comparing the mutational profiles of the matched tumor pairs, we outlined the mutational heterogeneity of HNSCCs (Figure 4A). In 5 clinically related tumor pairs, no mutations were detected at all. The remaining 46 matched tumor pairs allowed for analysis of mutational synonymy, revealing 92% (81/88) of the analyzed gene mutations to be concordantly present in the associated metastasis. One discordant mutation was found in *PTEN*. A different single somatic nucleotide variant was detected in the metastasis (c.316G>T) compared to its associated primary tumor (c.892C>T). Six mutations were exclusively detected in the primary tumor: *HRAS* (c.38G>T)*, TP53* (c.192_217del26)*, PIK3CA* (c.3140A>G)*, CDKN2A* (c.247C>G)*, MDM2* (c.158G>A). Additionally, 2 new mutations were detected in exclusively the metastasis: *SMAD4* (c.725C>G) and *ALK* (c.1588G>C). *TP53* mutations were detected in 43 matched tumor pairs and were concordantly present in the associated metastasis in 95% (41/43). Mutational concordance of *CDKN2A*, *HRAS* and *PIK3CA* was respectively 92% (11/12), 83% (5/6) and 83% (5/6). An overview of mutational profiles of associated primary tumors and metastases is shown in Supplementary Table S4.

Twenty-five associated pairs of primary tumors and recurrences were available for comparison (Figure 4B). In 2 matched tumor pairs, no mutations were detected. In the other 23 matched tumor pairs, 89% (33/37) of gene mutations found in the primary tumors were also found in the associated recurrences. No discordant mutations were detected. Four mutations were exclusively detected in the primary tumor: *PTEN* (c.892C>T)*, TP53* (c.192_217del26)*, HRAS* (c.34G>A)*, ERBB3* (c.1016G>A). Additionally, 5 new mutations were exclusively detected in the recurrent tumor sample: *FLT3* (c.2498C>G)*, CDKN2A* (c.172C>T)*, KIT* (c.1640A>G)*, TP53* (c.406delC)*, HRAS* (c.38G>T). *TP53* mutations were found concordant in 91% (21/23), 100% (4/4) in PIK3CA, 100% (2/2) in *CDKN2A*, and 50% (1/2) in *HRAS*. An overview of mutational profiles of associated primary tumors and recurrences is shown in Supplementary Table S5.

## DISCUSSION

Despite developments in diagnostic techniques and treatment, prognosis barely improved over the past few decades [8]. This is probably due to likelihood of recurrence (25-50%) after (chemo)radiotherapy and the lack of specific detection methods for recurrent disease [8, 13, 32]. And, although treatment options for recurrences depend on many different factors (e.g. initial disease stage, site of recurrence, previous treatment, expected quality of life), early detection of recurrences using biomarkers might contribute in improving disease outcome. Therefore, we think that the potential role of genetic biomarkers in the management of advanced stage HNSCC is of increasing importance. Although its potential role as a prognostic and predictive biomarker has been debated [33, 34], as well as its use for diagnosing recurrent or minimal residual disease during follow-up after treatment [35–38], *TP53* has, to our knowledge, not yet been proposed as a potential diagnostic biomarker for the early detection of locoregional metastases of HNSCC. Our data indicate that, using our 36-54 gene cancer panels, *TP53* is the most frequently mutated gene in HNSCC tumor samples. This was the case for primary tumors of all sites, as well as their associated recurrences and metastases. While previous studies of the mutational profile of HPV-negative HNSCC have shown prevalence rates of *TP53* mutations of 53%-78% [39, 40], these studies included either smaller sample sizes or were based on less sensitive whole exome sequencing (WES) data. More recent WES data from The Cancer Genome Atlas Network showed *TP53* mutations in 86% of 279 HPV-negative HNSCC samples, which is more consistent with our data [5]. Moreover, NGS data, based on clinical assays that were capable of deep coverage of cancer-relevant genes, found *TP53* mutations in 87% [4] and 81% [41] of HPV-negative HNSCCs.

In our study, *TP53* mutations were detected in only 64% of the primary OSCCs compared to 80% or more in other sites. Because HPV-positive OPSCCs and *TP53* mutations are known to be mutually exclusive in the majority of cases, in most studies similarly lowered *TP53* mutation rates are being found in OPSCCs [16, 42, 43]. In our study, this difference between OSCCs and other sites may also be due to the low number of HPV-positive OPSCC tumor samples. Another explanation may be the presence of epigenetic aberrations, copy number variations (CNV), or mutations in unknown genes that could have been more abundant in OSCCs than in tumors from other sites [44–46]. Also, differences in exon coverage of our gene panels could have affected the variability in mutation profiles of tumor samples, although the differences we found in *TP53* mutations cannot be fully proven because samples were not tested for all possible genetic aberrations and no statistical validation was performed. Additionally, statistical sampling bias could have affected sample sizes of HNSCC sites, since tumor type definition was primarily based on *TP53* clonality assessment. Moreover, because distinction between a recurrence and a second primary tumor is not always clear in clinical practice, the parameters of “time span” and “adjacent site”, used to determine whether a tumor is secondary or recurrent, may be interpreted differently in various studies. Re-evaluation of the available clinicopathological data did not yield any changes in *TP53* mutation prevalence rates.

Although not identified in hypopharyngeal tumors, we could confirm *CDKN2A* mutations as second most frequent mutations overall in HPV-negative HNSCCs. This is equivalent to findings in previous studies [4]. Compared to the literature, *HRAS* mutated less frequently in OSCC [5]. Furthermore, concordant with previous reports on *PIK3CA* [47], OSCCs contained *PIK3CA* mutations most frequently compared to other sites. Interestingly, concurrent PI3K pathway mutations such as PIK3R1/PIK3R2 were recently identified as being involved in HNSCC tumor progression. This supports the potential use of mutations in this oncogenic pathway as predictive biomarkers [48]. As expected, fewer *TP53* mutations were found in HNSCCs of non-drinking and non-smoking patients. All tumor subgroups exclusively related to alcohol and/or smoking contained the most *TP53* mutations. Furthermore, we found a relative increase of *PIK3CA* mutations in the non-smoking/non-drinking related group. NOTCH1 might also be a potential target as a diagnostic biomarker. NOTCH1 mutations are found in 14-20% of HNSCC and possibly play a role as early drivers in OSCC progression [2–4, 49, 50]. However, we found no aberrations in the NOTCH1 pathway. Aberrations were identified in the study of Agrawal *et al.*, in which tumor specimens were sequenced using WES based on assays that covered NOTCH1 exons 1-34. This difference in results might be because our gene panels only covered NOTCH1 exons 25, 27, and 37.

Clarifying tumor evolution genetically is of great importance, since tumor heterogeneity could seriously challenge the principle of using genetic mutations as (diagnostic) biomarkers [51, 52]. In order to use tumor specific mutations for quantifying purposes, it is essential to target mutations in ctDNA that are contained in both the primary tumor and its clonal expansions. Primary tumor biopsy (e.g. core needle, incision, or excision biopsy) carries the risk of incompletely depicting the mutational profile of primary tumor tissue due to intratumoral heterogeneity, a problem that increases with newly acquired mutations in clonal expansions. As a result, it can lead to tumor specific mutations being selected as biomarkers that are not present in ctDNA from clonal expansions. However, blood testing for these diagnostic biomarkers could identify mutations in *TP53* as well as those in other early driver genes that are extensively present in primary tumors and their clonal expansions.

Our data show that in most tumor pairs, mutations are concordant. This is largely consistent with the results of Hedberg et al. [53], who found that the primary tumor transmitted 86% of single somatic nucleotide variants identified in synchronous nodal metastases and 60% of those in recurrences. The relatively higher total amount of concordant mutations in associated recurrences compared to metastases in our study could be explained by the use of targeted gene panels instead of WES, which possibly impeded the detection of (unknown) driver genes that contribute to different pathways in tumor progression towards recurrences. Simultaneously, targeted sequencing could possibly have concealed intertumor heterogeneity, since we found comparable mutational synonymy rates of metastases and recurrences. On the other hand, our gene panels allowed for more sensitive sequencing compared to WES [57]. Thus, the small differences we found in mutational synonymy of *TP53* might suggest increased intertumor heterogeneity between primary tumors and recurrences compared to primary tumors and metastases. Another explanation for mutational discordance could be differences in tissue acquisition methods, because sometimes sequencing of primary tumor samples was performed on resection specimens, whereas sequencing of metastases was more often performed on (smaller) biopsies. This could have caused discordance due to intratumor heterogeneity.

Interestingly, the detection of two additional mutations (*SMAD4* and *ALK*) in two metastatic samples, might implicate these mutations to drive metastatic outgrowth, as these mutations, especially *SMAD4*, contribute to the downregulation of growth inhibitors and increased genomic instability [54]. Though, the number of mutations we found is not definite to draw conclusions. Furthermore, technical difficulties and flaws associated with performing NGS on FFPE material might have biased our results, as fragmented DNA originating from FFPE tissue challenges sequencing. Therefore, used NGS assays are adapted by using small amplicons facilitating shorter fragment sequencing. Also, fixation of tissue is known to potentially deaminate cytosines, possibly leading to more C>T or G>A base transitions [55]. However, recently performed validation of our gene panels revealed minimal FFPE induced DNA damage [56].

Despite their limitations, our findings provide useful information for developing new diagnostic strategies for HNSCC using targeted NGS panels that are easily accessible and capable of deep sequencing. Most investigated gene mutations were found concordantly mutated in the associated metastases and recurrences. Furthermore, *TP53* mutations are by far the most frequent. This suggests *TP53* mutations have potential value as diagnostic biomarkers in conjunction with subsequent ctDNA detection through liquid biopsy. By depicting these mutations in ctDNA using liquid biopsies, tumor remission after treatment could possibly be monitored non-invasively as compared to repeated biopsy for histological confirmation. This might complement current surveillance methods of clinical evaluation supported by flexible endoscopy and/or imaging such as PET-CT or diffusion weighted MRI, in order to increase accuracy for early detection of recurrent and/or metastatic HNSCC in the future.

## MATERIALS AND METHODS

### Data collection and analysis

We collected NGS sequencing data of all HNSCC samples, generated through TP53 clonality assessment on clinical request between the period of October 2013 and May 2015. Sequencing results from primary skin tumors of the head and neck region were not included. All samples on which sequencing was performed were formalin-fixed paraffin embedded (FFPE), after being obtained by surgical resection or tissue biopsy for diagnostic purposes between March 1992 to April 2015. Demographic and clinical data, including history of tobacco and alcohol use, were retrieved from hospital charts. Smoking and alcohol consumption habits were classified as previously described [58].

For analysis, samples were grouped and sorted by site of primary tumor (i.e. oral cavity, oropharynx, hypopharynx, larynx, miscellaneous), and tumor subtype (primary, metastasis, recurrence). Definition of tumor type (i.e. primary tumor, metastasis or recurrence) was mainly based on *TP53* clonality. If a clonal relationship could not be ruled out, we based subtype determination on clinical suspicion and date of incidence as described previously [58]. In the same manner, distinction was made between second primary tumors, metastases and recurrences. Samples were excluded from analysis if tumor subtype remained unclear. Also, samples of unknown anatomical origin and duplicates of sequencing results were excluded.

Sequencing results were retrieved from the nationwide network and registry of histo- and cytopathology in The Netherlands (PALGA). Descriptive analysis consisted of mutational prevalence, which was determined for each gene in primary tumors and for all tumor types. Because of the use of varying gene panels over time by our molecular diagnostics laboratory, all (average) percentages were weighed for differences in gene coverage of used gene panels. Furthermore, co-occurrence of gene mutations in primary tumors was determined. Mutational synonymy was assessed by comparing the genetic profiles of primary tumors or second primary tumors with matched locoregional and/or distant metastases, if present. Gene mutations within matched tumor pairs were considered concordant when alterations were identical in the primary tumor and its associated metastasis or recurrence. Associated tumor samples within each matched pair were consistently sequenced by the same gene panel.

### Molecular analysis

Clonality assessment was based on the presence of *TP53* mutations or similar loss of heterozygosity (LOH) profiles using short tandem repeats. Targeted NGS was performed using the Ion Torrent™ PGM platform (Thermo Fisher Scientific, Waltham, MA, USA) as previously described [56]. The following gene panels were used: CHPv2 (Thermo Fisher Scientific, Waltham, MA, USA), CHPv2+ (i.e. CHPv2 supplemented with several extra genes and amplicons) and OAPv2 [59]. Exact genes and exons sequenced are shown in Supplementary Table S6-8. References used for reporting gene mutations were Center for Personalized Cancer Treatment (CPCT, Utrecht, The Netherlands; http://www.cpct.nl), Catalogue Of Somatic Mutations in Cancer (COSMIC; http://cancer.sanger.ac.uk/cosmic), International Cancer Genome Consortium (ICGC; https://icgc.org/) and The Cancer Genome Atlas (TCGA; http://cancergenome.nih.gov/).

Sufficient coverage is reached when an amplicon was sequenced at least 500 times. Variants with an allele frequency below 1% were considered as background noise and were not reported. Variants with allele frequency between 1% and 5% were first discussed multidisciplinary before decision to report. Variants with allele frequencies above 5% were reported. The used assay was validated recently according to general rules for diagnostic laboratories through ISO certification [56]. Accordingly, minimum tumor percentage is set at 10%. Gene amplification was indicated when five or more amplicons showed a z-score of 5 or more [59]. Where NGS failed, additional Sanger sequencing of *TP53* exon 4-9 in forward and reverse directions was performed to allow for *TP53* clonality assessment. The Sanger sequencing products were analyzed on a 3730 DNA Analyzer (Applied Biosystems, Foster city, CA, USA). If sequencing failed altogether, samples were ultimately excluded from analysis.

## SUPPLEMENTARY TABLES




