# Supplementary material for: Targeted sequencing reveals TP53 as a potential diagnostic biomarker in the post-treatment surveillance of head and neck cancer
Source: Oncotarget. 2016 Aug 11;7(38):61575–86. doi: 10.18632/oncotarget.11196 (PMC5308673; doi:10.18632/oncotarget.11196)
Supplement: Supplementary file 1 [file oncotarget-07-61575-s001.pdf]

Targeted sequencing reveals *TP53* as a potential diagnostic biomarker in the post-treatment surveillance of head and neck cancer

SUPPLEMENTARY TABLES

Supplementary Table S1: Gene mutation count in primary HNSCC samples per site

	OPSCC (%)	OSCC (%)	HSCC (%)	LSCC (%)	Misc (%)
TP53	30 (81)	34 (64)	13 (81)	24 (86)	12 (80)
CDKN2A	1 (3)	7 (15)	0 (0)	3 (12)	3 (21)
PIK3CA	1 (3)	10 (22)	1 (7)	1 (4)	1 (7)
HRAS	3 (10)	5 (11)	1 (7)	0 (0)	1 (7)
FBXW7	1 (4)	1 (3)	0 (0)	1 (5)	0 (0)
FGFR3	1 (4)	1 (3)	0 (0)	0 (0)	1 (8)
SMAD4	0 (0)	0 (0)	0 (0)	1 (5)	1 (6)
ERBB4	0 (0)	0 (0)	0 (0)	0 (0)	1 (7)
BRAF	0 (0)	1 (2)	0 (0)	0 (0)	1 (6)
APC	0 (0)	0 (0)	0 (0)	0 (0)	1 (6)
ALK	0 (0)	0 (0)	0 (0)	1 (4)	1 (6)
GNAQ	0 (0)	0 (0)	0 (0)	1 (4)	1 (6)
PTEN	1 (3)	1 (2)	0 (0)	0 (0)	1 (6)
ATM	1 (4)	0 (0)	0 (0)	0 (0)	1 (8)
ERBB2	0 (0)	0 (0)	0 (0)	0 (0)	2 (13)
KIT	0 (0)	0 (0)	0 (0)	0 (0)	1 (6)
FLT3	0 (0)	1 (3)	0 (0)	0 (0)	0 (0)
RB1	0 (0)	1 (3)	1 (9)	0 (0)	1 (6)
KRAS	1 (3)	0 (0)	0 (0)	0 (0)	1 (6)
MTOR	0 (0)	1 (13)	0 (0)	0 (0)	1 (6)
SMARCB1	0 (0)	0 (0)	0 (0)	1 (5)	1 (6)
CDK4	1 (25)	0 (0)	1 (25)	0 (0)	1 (6)
ERBB3	0 (0)	0 (0)	1 (25)	0 (0)	1 (6)
MDM2	0 (0)	1 (7)	0 (0)	0 (0)	1 (6)
KDR	0 (0)	1 (2)	0 (0)	0 (0)	1 (6)
HNF1A	0 (0)	0 (0)	0 (0)	0 (0)	1 (8)

Supplementary Table S2: Gene mutation count in HNSCC samples per type

	Primary (%)	Recurrence (%)	Metastasis (%)
TP53	113 (76)	50 (82)	24 (83)
CDKN2A	12 (9)	11 (21)	4 (14)
PIK3CA	14 (11)	5 (10)	5 (18)
HRAS	9 (7)	3 (6)	2 (7)
FBXW7	4 (4)	1 (2)	1 (5)
FGFR3	2 (2)	1 (2)	0 (0)
BRAF	0 (0)	1 (2)	1 (4)
APC	0 (0)	1 (2)	2 (7)
PTEN	2 (2)	1 (2)	1 (4)
CDK4	1 (5)	0 (0)	1 (14)
ERBB4	0 (0)	0 (0)	1 (4)
ATM	2 (2)	1 (2)	0 (0)
ERBB2	1 (1)	1 (2)	0 (0)
SMAD4	0 (0)	1 (2)	0 (0)
FLT3	1 (1)	2 (4)	1 (4)
ALK	1 (1)	1 (2)	0 (0)
KRAS	1 (1)	3 (4)	0 (0)
MTOR	0 (0)	1 (9)	0 (0)
SMARCB1	0 (0)	0 (0)	0 (0)
GNAQ	1 (1)	0 (0)	0 (0)
ERBB3	1 (5)	0 (0)	0 (0)
MDM2	0 (0)	1 (9)	0 (0)
KDR	1 (1)	2 (4)	0 (0)
HNF1A	1 (1)	0 (0)	0 (0)
RB1	1 (1)	2 (5)	0 (0)
KIT	0 (0)	0 (0)	1 (4)

Supplementary Table S3: Absolute numbers of co-mutations between genes

See Supplementary File 1

Supplementary Table S4: Tumor pairs

See Supplementary File 1

Supplementary Table S5: Tumor pairs

PRIMARY				RECURRENCE			
Patient	Gene	Mutation	Type	Patient	Gene	Mutation	Type
1	HRAS	c.38G>A	MISSENSE	1	HRAS	c.38G>A	MISSENSE
1	FBX7	c.1153C>T	MISSENSE	1	FBX7	c.1153C>T	MISSENSE
2	TP53	c.586C>T	TRUNC	2	TP53	c.586C>T	TRUNC
3	TP53	c.438G>A	TRUNC	3	TP53	c.438G>A	TRUNC
4	TP53	c.574C>T	TRUNC	4	TP53	c.574C>T	TRUNC
4	ERBB4	c.513C>A	MISSENSE	4	ERBB4	c.513C>A	MISSENSE
5	TP53	c.490A>G	MISSENSE	5	TP53	c.490A>G	MISSENSE
5	TP53	c.489C>A	TRUNC	5	TP53	c.489C>A	TRUNC
5	PTEN	c.892C>T	TRUNC	5	PTEN	c.892C>T	TRUNC
6	TP53	c.473G>T	MISSENSE	6	TP53	c.473G>T	MISSENSE
6	TP53	c.192_217del26	INFRAME	7	TP53	c.809T>G	MISSENSE
7	TP53	c.809T>G	MISSENSE	8	TP53	c.560-2A>G	UNKNOWN
8	TP53	c.560-2A>G	UNKNOWN	8	CDKN2A	c.172C>T	TRUNC
9	PIK3CA	c.3140A>G	MISSENSE	9	PIK3CA	c.3140A>G	MISSENSE
10	N/A	N/A	N/A	10	N/A	N/A	N/A
11	TP53	c.742C>T	MISSENSE	11	TP53	c.742C>T	MISSENSE
12	TP53	c.413C>T	MISSENSE	11	KIT	c.1640A>G	MISSENSE
13	TP53	c.527G>A	MISSENSE	12	TP53	c.413C>T	MISSENSE
13	TP53	c.614A>G	MISSENSE	13	TP53	c.527G>A	MISSENSE
14	TP53	c.734G>A	MISSENSE	13	TP53	c.614A>G	MISSENSE
15	HRAS	c.34G>A	MISSENSE	14	TP53	c.734G>A	MISSENSE
15	PIK3CA	c.3140A>G	MISSENSE	15	HRAS	c.34G>A	MISSENSE
16	TP53	c.707A>G	MISSENSE	15	PIK3CA	c.3140A>G	MISSENSE
16	TP53	c.560del1	INFRAME	16	TP53	c.707A>G	MISSENSE
17	N/A	N/A	N/A	16	TP53	c.560del1	INFRAME
18	TP53	c.584T>C	MISSENSE	17	N/A	N/A	N/A
18	CDK4	c.693del1	INFRAME	18	TP53	c.584T>C	MISSENSE
18	ERBB3	c.1016G>A	MISSENSE	18	CDK4	c.693del1	INFRAME
19	TP53	c.475G>C	MISSENSE	19	TP53	c.475G>C	MISSENSE
20	TP53	c.916C>T	TRUNC	20	TP53	c.916C>T	TRUNC
21	TP53	c.610G>T	TRUNC	21	TP53	c.610G>T	TRUNC
21	CDKN2A	c.238C>T	TRUNC	21	CDKN2A	c.238C>T	TRUNC
22	TP53	c.637C>T	TRUNC	22	TP53	c.637C>T	TRUNC
23	TP53	c.706_708del3	INFRAME	23	TP53	c.706_708del3	INFRAME
23	PIK3CA	c.1353del14ins1	INFRAME	23	PIK3CA	c.1353del14ins1	INFRAME
23	CDKN2A	c.172C>T	TRUNC	23	CDKN2A	c.172C>T	TRUNC
24	TP53	c.181G>A	MISSENSE	24	TP53	c.181G>A	MISSENSE
24	PIK3CA	c.1633G>A	MISSENSE	24	HRAS	c.38G>T	MISSENSE
25	TP53	c.413C>T	MISSENSE	24	PIK3CA	c.1633G>A	MISSENSE
				25	TP53	c.413C>T	MISSENSE

Supplementary Table S6: Gene coverage of Ion AmpliSeq™ OncoAmp Panel v2:

AKT1	exon 1,3,6,9,13	ESR1	exon 3-7,10	MET	exon 2,5,14,16,19
ALK	exon 2,5,8,12,23,25	FGFR1	exon 2,4,7,9,14,18	MTOR	exon 2,12,24,46,58
BRAF	exon 3,7,11,15,18	FGFR2	exon 2,4,7,9,14,18	MYC	exon 1-3
CDH1*	exon 1-16	FGFR4	exon 5,11,16,18	MYCN	exon 2,3
CDK4	exon 1-8	FLT3	exon 5,11,14,16,20	NRAS	exon 2,3
CDK6	exon 1-6,8	GNAS	exon 8	PDGFRA	exon 6,12,14,16,18,23
CDKN2A	exon 2	GNAQ	exon 5	PIK3CA	exon 1,4,7,9,13,20
CTNNB1	exon 3	HRAS	exon 2,3,5	PTEN*	exon 1-9
EGFR*	exon 1-28	KDR	exon 6,7,11,19,21,26,27,30	RET	exon 10,11,13,15,17,18
ERBB2	exon 5,11,22-24,30	KIT	exon 2,10,11,13-15,17,18	TOP2A	exon 2,8,14,22,28,34
ERBB3	exon 5,9,13,22,27	KRAS	exon 2-5	TP53*	exon 2-10
ERBB4	exon 3,4,6-8,15,22,27	MDM2*	exon 1-11	VHL*	exon 1-3

* Full transcript covered in this gene panel. Of the remaining genes, only regions that are frequently mutated were sequenced instead of the whole coding sequence. Amplifications were able to be detected in genes covered by at least 5 amplicons.

Supplementary Table S7: Gene coverage of Ion AmpliSeq™ Cancer Hotspot Panel v2

ABL1	exon 4-7	EZH2	exon 16	JAK2	exon 14	PTEN	exon 3,5-8
AKT1	exon 3, 6	FBXW7	exon 5,8-11	JAK3	exon 4,13,16	PTPN11	exon 3,13
ALK	exon 23,25	FGFR1	exon 4,7	KDR	exon 6,7,11,19,21,26,30	RB1	exon 4,6,10,11,14,17
APC	exon 14	FGFR2	exon 5,7,10	KIT	exon 2,9-11,13-15,17,18	RET	exon 10,11,13,15,16
ATM	exon 8,9,12,17,26,34,35,36,39,50,54,55,56,59,61,63	FGFR3	exon 7,9,14,16,18	KRAS	exon 2-4	SMAD4	exon 3-6,8-12
BRAF	exon 11,15	FLT3	exon 11,14,16,20	MET	exon 2,11,14,16,19	SMARCB1	exon 2,4,5
CDH1	exon 3,8,9	GNA11	exon 5	MLH1	exon 12	SMO	exon 3,5,6,9,11
CDKN2A	exon 2	GNAS	exon 8,9	MPL	exon 10	SRC	exon 14
CSF1R	exon 7,22	GNAQ	exon 5	NOTCH1	exon 25,27,37	TP53	exon 3-8,10
CTNNB1	exon 3	HNF1A	exon 3,4	NPM1	exon 11	VHL	exon 1-3
EGFR	exon 3,7,15,18-21	HRAS	exon 2,3	NRAS	exon 2-4		
ERBB2	exon 22-24	IDH1	exon 4	PDGFRA	exon 12,14,15,18		
ERBB4	exon 3-5,7-9,15,23	IDH2	exon 4	PIK3CA	exon 2,5,7-10,14,19,21		

Supplementary Table S8: Gene coverage of Ion AmpliSeq™ Cancer Hotspot Panel v2+:

ABL1	exon 4-7	FGFR2	exon 5,7,10	MYD88	exon 5
AKT1	exon 3,6	FGFR3	exon 7,9,14,16,18	NOTCH1	exon 25,27,37
ALK	exon 22-25		NPM1		exon 11
APC	exon 14	GNA11	exon 5	NRAS	exon 2-4
ARAF	exon 6	GNAS	exon 8,9	PDGFRA	exon 12,14,15,18
ATM	exon 8,9,12,17,26,34,35, 36,39,50,54,55,56,59,61,63	GNAQ	exon 5	PIK3CA	exon 1,4,6,7,9,13, 18,20
BRAF	exon 11,15	HNF1A	exon 3,4	PTEN	exon 3,5-8
CALR	exon 9	HRAS	exon 2,3	PTPN11	exon 3,13
CDH1	exon 3,8,9	IDH1	exon 4	RB1	exon 4,6,10,11,14,17
CDKN2A	exon 2	IDH2	exon 4	RET	exon 10,11,13,15,16
CRAF	exon 6	JAK2	exon 14	SMAD4	exon 3-6,8-12
CSF1R	exon 7, 22	JAK3	exon 4,13,16	SMARCB1	exon 2,4,5
CTNNB1	exon 3	KDR	exon 6,7,11,19,21,26,30	SMO	exon 3,5,6,9,11
EGFR	exon 3,7,15,18-21	KIT	exon 2,9-11,13- 15,17,18	SRC	exon 14
ERBB2	exon 19-24	KRAS	exon 2-4	STK11	exon 1,4,6,8
ERBB4	exon 3-5,7-9,15,23	MDM2	exon 6-9	TP53	exon 2-10
EZH2	exon 16	MET	exon 2,11,14,16,19	VHL	exon 1-3
FBXW7	exon 5,8-11		MLH1		exon 12
FGFR1	exon 4,7		MPL		exon 10

*This assay covers all hotspot regions, but does not give information about the complete coding sequence of depicted genes.
**This assay is not yet officially validated for the detection of amplifications
