# Supplementary material for: Targeted sequencing reveals TP53 as a potential diagnostic biomarker in the post-treatment surveillance of head and neck cancer
Source: Oncotarget. 2016 Aug 11;7(38):61575–86. doi: 10.18632/oncotarget.11196 (PMC5308673; doi:10.18632/oncotarget.11196)
Supplement: Supplementary file 2 [file oncotarget-07-61575-s002.docx]

| Genes | HNF1A | KDR | MDM2 | ERBB3 | CDK4 | MTOR | KRAS | FLT3 | KIT | ERBB2 | FGFR3 | BRAF | ERBB4 | PIK3CA | HRAS | GNAQ | ALK | SMAD4 | SMARCB1 | RB1 | ATM | PTEN | FBXW7 | CDKN2A | APC |
| --- | --- | --- | --- | --- | --- | --- | --- | --- | --- | --- | --- | --- | --- | --- | --- | --- | --- | --- | --- | --- | --- | --- | --- | --- | --- |
| TP53 | - | 3 | 2 | 1 | 3 | 2 | 4 | 4 | 1 | 2 | 4 | - | 2 | 15 | 8 | 1 | 2 | 2 | 1 | 4 | 2 | 3 | 2 | 27 | 3 |
| APC | - | - | - | - | - | - | - | - | - | - | - | - | - | 1 | 1 | - | - | - | - | - | - | - | - | - | - |
| CDKN2A | - | 3 | 2 | - | - | - | - | 3 | 2 | 2 | 3 | - | - | 5 | 2 | - | - | - | - | 2 | 2 | - | 2 | - | - |
| FBXW7 | - | - | - | - | - | - | - | - | - | - | - | - | - | 3 | 2 | - | - | - | - | - | 1 | - | - | - | - |
| PTEN | - | - | - | - | - | - | - | - | - | - | - | - | - | - | - | - | - | 1 | - | - | 1 | - | - | - | - |
| ATM | - | - | - | - | - | - | - | - | - | 2 | 2 | - | - | - | 2 | - | - | - | - | - | - | - | - | - | - |
| RB1 | - | - | - | - | - | - | - | - | - | - | - | - | - | - | - | - | - | - | - | - | - | - | - | - | - |
| SMARCB1 | - | - | - | - | - | - | - | - | - | - | - | - | - | - | - | - | - | - | - | - | - | - | - | - | - |
| SMAD4 | - | - | - | - | - | - | - | - | - | - | 1 | - | - | - | - | 1 | 1 | - | - | - | - | - | - | - | - |
| ALK | - | - | - | - | - | - | - | - | - | - | - | - | - | - | - | 1 | - | - | - | - | - | - | - | - | - |
| GNAQ | - | - | - | - | - | - | - | - | - | - | - | - | - | - | - | - | - | - | - | - | - | - | - | - | - |
| HRAS | - | - | - | - | 1 | - | - | - | - | 2 | 2 | - | - | 6 | - | - | - | - | - | - | - | - | - | - | - |
| PIK3CA | - | - | - | - | - | - | - | - | - | - | - | 1 | - | - | - | - | - | - | - | - | - | - | - | - | - |
| ERBB4 | - | - | - | - | - | - | - | - | 1 | - | 1 | - | - | - | - | - | - | - | - | - | - | - | - | - | - |
| BRAF | - | - | - | - | - | - | - | - | - | - | 1 | - | - | - | - | - | - | - | - | - | - | - | - | - | - |
| FGFR3 | - | - | - | - | - | - | - | - | - | 2 | - | - | - | - | - | - | - | - | - | - | - | - | - | - | - |
| ERBB2 | - | - | - | - | - | - | - | - | - | - | - | - | - | - | - | - | - | - | - | - | - | - | - | - | - |
| KIT | - | - | - | - | - | - | - | - | - | - | - | - | - | - | - | - | - | - | - | - | - | - | - | - | - |
| FLT3 | - | 3 | 2 | - | - | - | - | - | - | - | - | - | - | - | - | - | - | - | - | - | - | - | - | - | - |
| KRAS | - | - | - | - | - | - | - | - | - | - | - | - | - | - | - | - | - | - | - | - | - | - | - | - | - |
| MTOR | - | - | - | - | - | - | - | - | - | - | - | - | - | - | - | - | - | - | - | - | - | - | - | - | - |
| CDK4 | - | - | - | 1 | - | - | - | - | - | - | - | - | - | - | - | - | - | - | - | - | - | - | - | - | - |
| ERBB3 | - | - | - | - | - | - | - | - | - | - | - | - | - | - | - | - | - | - | - | - | - | - | - | - | - |
| MDM2 | - | 2 | - | - | - | - | - | - | - | - | - | - | - | - | - | - | - | - | - | - | - | - | - | - | - |
| KDR | - | - | - | - | - | - | - | - | - | - | - | - | - | - | - | - | - | - | - | - | - | - | - | - | - |

**Table S3:** Absolute numbers of co-mutations between genes

**Table S4:** Tumor pairs

| PRIMARY TUMOR | | | | METASTASIS | | | |
| --- | --- | --- | --- | --- | --- | --- | --- |
|  |  |  |  |  |  |  |  |
| Pair | Gene | Mutation | Type | Pair | Gene | Mutation | Type |
| 1 | HRAS | c.181C>A | MISSENSE | 1 | HRAS | c.181C>A | MISSENSE |
| 1 | PIK3CA | c.1633G>A | MISSENSE | 1 | PIK3CA | c.1633G>A | MISSENSE |
| 2 | HRAS | c.181C>A | MISSENSE | 2 | HRAS | c.181C>A | MISSENSE |
| 2 | PIK3CA | c.1633G>A | MISSENSE | 2 | PIK3CA | c.1633G>A | MISSENSE |
| 3 | TP53 | c.536A>G | MISSENSE | 3 | TP53 | c.536A>G | MISSENSE |
| 4 | N/A | N/A | N/A | 4 | N/A | N/A | N/A |
| 5 | TP53 | c.838A>G | MISSENSE | 5 | TP53 | c.838A>G | MISSENSE |
| 5 | HRAS | c.38G>T | MISSENSE | 6 | N/A | N/A | N/A |
| 6 | N/A | N/A | N/A | 7 | TP53 | c.306C>A | MISSENSE |
| 7 | TP53 | c.306C>A | MISSENSE | 7 | TP53 | c.310C>T | TRUNC |
| 7 | TP53 | c.310C>T | TRUNC | 8 | TP53 | c.490A>G | MISSENSE |
| 8 | TP53 | c.490A>G | MISSENSE | 8 | TP53 | c.489C>A | TRUNC |
| 8 | TP53 | c.489C>A | TRUNC | 8 | SMAD4 | c.725C>G | TRUNC |
| 8 | PTEN | c.892C>T | TRUNC | 8 | PTEN | c.316G>T | TRUNC |
| 9 | TP53 | c.659A>G | MISSENSE | 9 | N/A | N/A | N/A |
| 9 | PIK3CA | c.3140A>G | MISSENSE | 10 | TP53 | c.733G>A | MISSENSE |
| 9 | CDKN2A | c.247C>G | MISSENSE | 10 | HRAS | c.172C>T | TRUNC |
| 10 | TP53 | c.733G>A | MISSENSE | 11 | TP53 | c.473G>T | MISSENSE |
| 10 | HRAS | c.172C>T | TRUNC | 12 | TP53 | c.799C>G | MISSENSE |
| 11 | TP53 | c.473G>T | MISSENSE | 12 | HRAS | c.183G>T | MISSENSE |
| 11 | TP53 | c.192_217del26 | INFRAME | 12 | CDKN2A | c.238C>T | TRUNC |
| 12 | TP53 | c.799C>G | MISSENSE | 12 | FGFR3 | c.1108G>A | MISSENSE |
| 12 | HRAS | c.183G>T | MISSENSE | 12 | ATM | c.9124C>T | MISSENSE |
| 12 | CDKN2A | c.238C>T | TRUNC | 12 | ERBB2 | c.2593-2594GG>AA | MISSENSE |
| 12 | FGFR3 | c.1108G>A | MISSENSE | 13 | TP53 | c.818G>T | MISSENSE |
| 12 | ATM | c.9124C>T | MISSENSE | 14 | TP53 | c.659A>G | MISSENSE |
| 12 | ERBB2 | c.2593-2594GG>AA | MISSENSE | 14 | PIK3CA | c.1624G>A | MISSENSE |
| 13 | TP53 | c.818G>T | MISSENSE | 15 | TP53 | c.730G>T | MISSENSE |
| 14 | TP53 | c.659A>G | MISSENSE | 15 | CDKN2A | c.192_194delGCT | MISSENSE |
| 14 | PIK3CA | c.1624G>A | MISSENSE | 15 | FBXW7 | c.1273C>G | MISSENSE |
| 15 | TP53 | c.730G>T | MISSENSE | 16 | TP53 | c.583delA | INFRAME |
| 15 | CDKN2A | c.192_194delGCT | MISSENSE | 17 | TP53 | c.920-1G>T | UNKNOWN |
| 15 | FBXW7 | c.1273C>G | MISSENSE | 18 | N/A | N/A | N/A |
| 16 | TP53 | c.583delA | INFRAME | 19 | TP53 | c.841_842del2 | INFRAME |
| 17 | TP53 | c.920-1G>T | UNKNOWN | 19 | RB1 | c.2107-1G>C | UNKNOWN |
| 18 | N/A | N/A | N/A | 20 | N/A | N/A | N/A |
| 19 | TP53 | c.841_842del2 | INFRAME | 21 | TP53 | c.743G>A | MISSENSE |
| 19 | RB1 | c.2107-1G>C | UNKNOWN | 22 | N/A | N/A | N/A |
| 20 | N/A | N/A | N/A | 23 | TP53 | c.105del5 | INFRAME |
| 21 | TP53 | c.743G>A | MISSENSE | 23 | KRAS | c.38G>A | MISSENSE |
| 22 | TP53 | c.379T>C | MISSENSE | 24 | TP53 | c.396G>T | MISSENSE |
| 23 | TP53 | c.105del5 | INFRAME | 25 | N/A | N/A | N/A |
| 23 | KRAS | c.38G>A | MISSENSE | 26 | TP53 | c.551delA | INFRAME |
| 24 | TP53 | c.396G>T | MISSENSE | 26 | CDKN2A | c.205G>T | TRUNC |
| 25 | N/A | N/A | N/A | 27 | TP53 | c.592G>T | TRUNC |
| 26 | TP53 | c.551delA | INFRAME | 28 | TP53 | c.524G>A | MISSENSE |
| 26 | CDKN2A | c.205G>T | TRUNC | 28 | CDKN2A | c.172C>T | TRUNC |
| 27 | TP53 | c.592G>T | TRUNC | 28 | RB1 | c.1698+6T>G | UNKNOWN |
| 28 | TP53 | c.524G>A | MISSENSE | 29 | TP53 | c.225-35G>C | UNKNOWN |
| 28 | CDKN2A | c.172C>T | TRUNC | 30 | TP53 | c.225-35G>C | UNKNOWN |
| 28 | RB1 | c.1698+6T>G | UNKNOWN | 31 | TP53 | c.742C>T | MISSENSE |
| 29 | TP53 | c.225-35G>C | UNKNOWN | 31 | TP53 | c.764_765delTCinsAT | MISSENSE |
| 30 | TP53 | c.225-35G>C | UNKNOWN | 31 | CDKN2A | c.172C>T | TRUNC |
| 31 | TP53 | c.742C>T | MISSENSE | 32 | TP53 | c.329G>T | MISSENSE |
| 31 | TP53 | c.764_765delTCinsAT | MISSENSE | 32 | TP53 | c.637C>T | TRUNC |
| 32 | TP53 | c.329G>T | MISSENSE | 33 | TP53 | c.505_506insT | INFRAME |
| 32 | TP53 | c.637C>T | TRUNC | 33 | TP53 | c.769C>G | MISSENSE |
| 33 | TP53 | c.505_506insT | INFRAME | 33 | MTOR | c.34G>A | MISSENSE |
| 33 | TP53 | c.769C>G | MISSENSE | 34 | TP53 | c.101dupC | INFRAME |
| 33 | MTOR | c.34G>A | MISSENSE | 35 | TP53 | c.818G>A | MISSENSE |
| 34 | TP53 | c.101dupC | INFRAME | 35 | PIK3CA | c.1624G>A | MISSENSE |
| 35 | TP53 | c.818G>A | MISSENSE | 36 | TP53 | c.700T>C | MISSENSE |
| 35 | PIK3CA | c.1624G>A | MISSENSE | 36 | ALK | c.1588G>C | MISSENSE |
| 36 | TP53 | c.700T>C | MISSENSE | 37 | TP53 | c.700T>C | MISSENSE |
| 37 | TP53 | c.700T>C | MISSENSE | 38 | TP53 | c.637C>T | TRUNC |
| 38 | TP53 | c.637C>T | TRUNC | 39 | TP53 | c.733G>A | MISSENSE |
| 39 | TP53 | c.733G>A | MISSENSE | 39 | CDKN2A | c.172C>T | TRUNC |
| 40 | TP53 | c.733G>A | MISSENSE | 40 | TP53 | c.733G>A | MISSENSE |
| 40 | CDKN2A | c.172C>T | TRUNC | 40 | CDKN2A | c.172C>T | TRUNC |
| 41 | TP53 | c.476delC | INFRAME | 41 | TP53 | c.476delC | INFRAME |
| 42 | TP53 | c.743G>C | MISSENSE | 42 | TP53 | c.743G>C | MISSENSE |
| 43 | TP53 | c.457_460del4 | INFRAME | 43 | TP53 | c.457_460del4 | INFRAME |
| 43 | TP53 | c.375_375+1del2ins2 | UNKNOWN | 43 | TP53 | c.375_375+1del2ins2 | UNKNOWN |
| 44 | TP53 | c.734G>A | INFRAME | 44 | TP53 | c.734G>A | INFRAME |
| 44 | CDKN2A | c.151-1G>A | UNKNOWN | 44 | CDKN2A | c.151-1G>A | UNKNOWN |
| 44 | FLT3 | c.505A>G | MISSENSE | 44 | FLT3 | c.505A>G | MISSENSE |
| 44 | MDM2 | c.158G>A | MISSENSE | 44 | KDR | c.2630G>A | MISSENSE |
| 44 | KDR | c.2630G>A | MISSENSE | 45 | HRAS | c.181C>A | MISSENSE |
| 45 | HRAS | c.181C>A | MISSENSE | 45 | PIK3CA | c.1633G>A | MISSENSE |
| 45 | PIK3CA | c.1633G>A | MISSENSE | 46 | TP53 | c.743G>A | MISSENSE |
| 46 | TP53 | c.743G>A | MISSENSE | 47 | TP53 | c.396G>T | MISSENSE |
| 47 | TP53 | c.396G>T | MISSENSE | 48 | TP53 | c.700T>C | MISSENSE |
| 48 | TP53 | c.700T>C | MISSENSE | 49 | TP53 | c.733G>A | MISSENSE |
| 49 | TP53 | c.733G>A | MISSENSE | 49 | CDKN2A | c.172C>T | TRUNC |
| 49 | CDKN2A | c.172C>T | TRUNC | 50 | TP53 | c.734G>A | MISSENSE |
| 50 | CDKN2A | c.151-1G>A | UNKNOWN | 50 | CDKN2A | c.151-1G>A | UNKNOWN |
| 50 | FLT3 | c.505A>G | MISSENSE | 50 | FLT3 | c.505A>G | MISSENSE |
| 50 | MDM2 | c.158G>A | MISSENSE | 50 | MDM2 | c.158G>A | MISSENSE |
| 50 | KDR | c.2630G>A | MISSENSE | 50 | KDR | c.2630G>A | MISSENSE |
| 51 | TP53 | c.734G>A | MISSENSE | 51 | TP53 | c.734G>A | MISSENSE |
| 51 | CDKN2A | c.151-1G>A | UNKNOWN | 51 | CDKN2A | c.151-1G>A | UNKNOWN |
| 51 | FLT3 | c.505A>G | MISSENSE | 51 | FLT3 | c.505A>G | MISSENSE |
| 51 | MDM2 | c.158G>A | MISSENSE | 51 | MDM2 | c.158G>A | MISSENSE |
| 51 | KDR | c.2630G>A | MISSENSE | 51 | KDR | c.2630G>A | MISSENSE |
